# Pan-dent-emic: a dentist’s dilemma in the COVID-19 era

**DOI:** 10.1186/s13037-021-00282-w

**Published:** 2021-02-14

**Authors:** Nidhi Manaktala, Swati Pralhad, Roma M

**Affiliations:** 1 Department of Oral Pathology and Microbiology Manipal College of Dental Sciences, Manipal Academy of Higher Education, Karnataka 575001 Mangalore, India; 2 Department of Periodontology, Manipal College of Dental Sciences, Manipal Academy of Higher Education, Karnataka 575001 Mangalore, India; 3 Department of Conservative Dentistry and Endodontics Manipal College of Dental Sciences, Manipal Academy of Higher Education, Karnataka 575001 Mangalore, India

**Keywords:** Corona virus, COVID-19, Dentistry SARS- COV 2

## Abstract

The aftermath of the COVID-19 pandemic has been unfathomable. It has affected every sector within health care industry with dentistry being one of the worst hits. Not only has it impacted the dental practice, the field of dental education has been affected as well. There has been loss in terms of delayed to no treatments, finances, psychology and most importantly breaks in ongoing education and research practices. The present article attempts to explain the dilemma of the current situation from a dentist’s perspective. Since, the effects of the contagion are seen across each level of dentistry, the current situation can truly be termed as a “Pan-dent-emic”.

The global pandemic spread by corona virus has apprehended the entire world and has created unprecedented concerns among health care personnel and general public alike. It has not only affected people’s physical health, but also their mental health. Despite various efforts to prevent the pandemic spread, the flare-up of the disease is still mounting due to the community spread in every area. Additionally, the contagion has affected Dentistry not only in terms of practice management, but also at the most basic level of imparting dental education to students. While on one end of the spectrum, dental practice has been nearly stalled due to the fact that dental care professionals are at maximum risk of COVID-19 (SARS-CoV-2) infection, there also have been unforeseen changes in teaching practices and curriculum to make pedagogy more efficient in these times.

In an effort to combat COVID-19 infection, the world has conformed to the age-old Latin phrase “Aut inveniam viam aut faciam”, wherein reformed practices have been instituted to provide dental care and education in these trying times. The standstill in dental practice having led to delayed diagnoses and treatment has had many implications on patients. Nevertheless, dental practice management has striven endlessly to redeem itself using safety measures like tele-triages, screening of patients with questionnaires and digital thermometers; use of PPE (personal protective equipment) by dentist and auxiliaries and extensive sanitization during dental procedures [1]. The practice of tele-consultation, time-bound appointments and following social distancing is already explained to patients before entering clinics so as to prevent overcrowding and cross contamination in clinical settings [2]. However, the psycho-economic setbacks incurred by the dental practitioner would take a long time to heal.

The education counterpart, however, has faced much indiscretion due to sudden changes in teaching practices. Recently, a term “covido-pedago-phobia” coined by Eachempati and Ramanaryan (2020) elucidated the quandary of timid ‘digital immigrant’ teachers who might experience mental paucity and reluctance while transitioning towards a more tech-savvy mode of teaching. They also discussed the impact of these modalities on students, the most important being the lack of social interaction leading to students becoming socially inept, which may undermine their ability to face challenges in the future [3]. Furthermore, in academic institutions, there has also been a simultaneous halt on research, biopsy and community services which has led to loss of time, finances along with an incumbent personal duress [4].

Overall, the COVID-19 crisis has led to an economic, psycho-social and physical impact, encompassing all aspects of dentistry. Hence, the present scenario can rightfully be termed as a “Pan-dent-emic”. However, as they say, ‘every cloud has a silver lining’; the advent of this pandemic has brought in a lot of positives as well. The dental community has never been more connected than present through webinars, online conferences and virtual continuing dental education programs [4]. There has also been a surge in medical/dental innovations wherein young researchers are working on applications to increase awareness via innovative digital mobile applications, creating devices to reduce and contain aerosols during dental procedures and much more [5]. The use of Whatsapp and other digital platforms in teaching, public health and reporting services have truly given rise to what we have always known as Tele-dentistry [6, 7].

On a general note, there has been a drastic decrease in respiratory illnesses with a natural cleansing of the environment [8]. Even when people are suffering from mental health issues, a study conducted by Barzilay (2020) indicated the signs of altruism in people’s COVID-19 worries. They reported that increased resilience was paramount in reducing rates of anxiety and depression during the pandemic [9]. On a positive note, as and when the stabilization takes place, we as members of the oral health community must show resilience and ought to be grateful; and be cognizant that the old and the new normal must go hand in hand to truly bring back things to a state of equilibrium.

## Data Availability

Data sharing is not applicable to this article as no datasets were generated or analyzed during the current study.

## Abbreviations

SARS-CoV: Severe Acute Respiratory Syndrome Coronavirus
COVID-19: Coronavirus disease

## References

[1] Patini R (2020). How to face the post-SARS-CoV-2 outbreak era in private dental practice: current evidence for avoiding cross-infections. J Int Soc Prev Community Dent.

[2] Ludovichetti FS, Signoriello AG, Stellini E, Mazzoleni S. COVID-19, rules of conduct for dental care in children during pandemic. Minerva Stomatol. 2020. 10.23736/S0026-4970.20.04419-2. Epub ahead of print. PMID: 32744445. 32744445

[3] Eachempati P, Ramnarayan K (2020). Covido-pedago-phobia. Med Educ.

[4] Wu DT, Wu KY, Nguyen TT, Tran SD. The impact of COVID-19 on dental education in North America-Where do we go next? Eur J Dent Educ. 2020. 10.1111/eje.12561. Epub ahead of print. PMID: 32654328; PMCID: PMC7404882. PMC7404882 32654328

[5] Dente SMR, Hashimoto S (2020). COVID-19: A pandemic with positive and negative outcomes on resource and waste flows and stocks. Resour Conserv Recycl.

[6] Das R, Manaktala N, Bhatia T, Agarwal S, Natarajan S, Lewis AJ, Yellapurkar S (2020). Efficiency of mobile video sharing application (WhatsApp®) in live field image transmission for telepathology. J Med Syst..

[7] Gueiros LA, Melo TS, Carrard VC. A simple tool to a complex reality-WhatsApp use in a developing country during COVID-19 pandemic. Oral Dis. 2020.10.1111/odi.13495. Epub ahead of print. PMID: 32558069. 10.1111/odi.13495 32558069

[8] Nelson B (2020). The positive effects of covid-19. BMJ..

[9] Barzilay R, Moore TM, Greenberg DM, Di Domenico GE, Brown LA, White LK, Gur RC, Gur RE (2020). Resilience COVID-19-related stress, anxiety and depression during the pandemic in a large population enriched for healthcare providers. Transl Psychiatry..

